# The effect of nutrient enrichment on the growth, nucleic acid concentrations, and elemental stoichiometry of coral reef macroalgae

**DOI:** 10.1002/ece3.330

**Published:** 2012-07-16

**Authors:** Ruth Reef, John M Pandolfi, Catherine E Lovelock

**Affiliations:** 1 School of Biological Sciences, The University of QueenslandSt Lucia, QLD, 4072, Australia; 2 Centre for Marine Science, School of Biological Sciences and ARC Centre of Excellence for Coral Reef Studies, The University of QueenslandSt Lucia, QLD, 4072, Australia

**Keywords:** Ecostoichiometry, eutrophication, growth rate hypothesis, luxury uptake, tropical algae

## Abstract

The growth rate hypothesis (GRH) links growth rates with organism elemental stoichiometry. Support for the GRH was found for many animal species, but less so for plants. This is the first study to test the GRH in macroalgae. Tropical coral reef macroalgae from three lineages, *Caulerpa serrulata* (Chlorophyta), *Laurencia intricata* (Rhodophyta), and *Sargassum polyphyllum* (Phaeophyceae) were grown enriched with nitrogen or phosphorous and under control conditions at Heron Island on the Great Barrier Reef, Australia. Growth rate, photosynthesis, nucleic acid composition, and elemental stoichiometry were measured. Nutrient enrichment had positive effects on photosynthetic rates and on investment in RNA. However, growth rate was not correlated with either photosynthetic rates or RNA content; thus, we did not find support for the GRH in tropical macroalgae. Macroalgae, especially *L. intricata*, accumulated P to very high levels (>0.6% of dry weight). The growth rate response to tissue P concentrations was unimodal. Above 0.21%, P accumulation had negative effects on growth. Nitrogen was not stored, but evidence of futile cycling was observed. The capacity to store large amounts of P is probably an adaptation to the low and patchy nutrient environment of the tropical oceans.

## Introduction

Nutrient availability is among the most important variables determining plant productivity and growth. In order to attain maximum growth, nutrients need to be supplied or acquired by the plant in a stoichiometrically balanced manner (Aerts and Chapin 2000). The elemental stoichiometry of plant tissues (mainly carbon (C), nitrogen (N), and phosphorous (P)) is linked to plant growth because of the constrained chemistry of the molecules important for growth such as DNA, proteins, and ribosomes (Elser et al. 2000b). Plants can display significant variations in C:N:P stoichiometry depending on light and CO_2_ availability, nutrient supply, and phylogeny. How these factors affect growth rates has been the topic of extensive scientific debate (see reviews by Ågren 2008; Sardans et al. 2011).

Plants are also able to store significant amounts of nutrients as reserves for future growth (Chapin et al. 1990), which can significantly alter stoichiometric ratios and stoichiometric homeostasis. In aquatic environments, highly constrained C:N:P ratios have been observed, compared with terrestrial plants (Elser et al. 2000a), possibly due to the more uniform distribution of nutrients in the water column in comparison with soils, and also because of the overall lower investment of C in plant structure in aquatic plants. A large-scale analysis of nutrient concentrations in tissues of aquatic plants revealed that while nutrient concentrations differ greatly among plant species, C:N:P ratios were relatively constrained (Duarte 1992). The elemental composition of a plant is of significance not only for growth and reproduction but also for rates of decomposition (Güsewell and Gessner 2009) and the nutritional value to consumers (Sterner and Hessen 1994).

The growth rate hypothesis proposes that growth rate is dependent on resource allocation (mainly P but also N) to ribosomal RNA, such that faster growing organisms require an overall greater P content associated with more rRNA and increased capacity for protein synthesis. Variation in P content (or C:N:P stoichiometry) is thus attributed to differences in RNA content that reflect different capacities for growth. The GRH has been tested for a variety of organisms (reviewed in Elser et al. 2003), including higher plants (Lovelock et al. 2007; Reef et al. 2010). Strong support for the GRH was found for bacteria (Kennell and Magasanik 1962), many animal species (e.g., Church and Robertson 1966; Buckley 1984; Wagner et al. 1998), and phytoplankton (Dortch et al. 1983). However, in higher plants (Reef et al. 2010) and marine microalgae (Flynn et al. 2010), support is not as strong, mostly due to their ability to store considerable amounts of nutrients (Matzek and Vitousek 2009; Flynn et al. 2010), especially under nutrient-limited conditions (Ågren 2004). This study provides the first test for the growth rate hypothesis in macroalgae.

Carbonate-rich tropical systems are generally believed to be P limited (Lapointe et al. 1992). Carbonate sediments in oligotrophic waters are strong sinks for P (McGlathery et al. 1994) resulting in less P available for algal uptake. Nitrogen is fixed on coral reefs at high rates (Mague and Holm-Hanson 1975; Potts and Whitton 1977; Shashar et al. 1994), and coral reefs are known to export N to surrounding waters (Hanson and Gundersen 1976). The average N:P ratios of benthic marine plants is 30:1 (Atkinson and Smith 1983), suggesting departure from the Redfield Ratio (Redfield 1958) and thus, potential P limitation. However, coral reef macroalgae show mixed responses to nutrient enrichment. Nitrogen has been shown to enhance growth in some coral reef algae, while increased P availability enhanced growth in other macroalgal species from the same location (Smith et al. 1981). In other species, growth was not affected or was even inhibited by nutrient enrichment (Littler et al. 1991). Simultaneous N and P limitation has been recorded for some Great Barrier Reef (GBR) macroalgae (Schaffelke and Klumpp 1998).

Macroalgae are an important component of both healthy and degraded coral reefs through their contribution to reef biodiversity, structure, primary productivity, and nitrogen fixation (see review by McCook 1999). However, when macroalgal abundance is enhanced to a great degree, this could have negative impacts on the health of coral reef ecosystems (Hughes 1994). Eutrophication is often cited as a major cause for the increase in macroalgae biomass and concurrent coral decline observed on some coral reefs (for review see Szmant 2002). However, how coral reef macroalgae respond to elevated nutrients is unclear. Although a few studies show a direct positive growth response of coral reef macroalgae to eutrophication events (Smith et al. 1981; Genin et al. 1995), there are other studies that show reduced growth rates of macroalgae following eutrophication (e.g., Drew 1983; McCook 1996; McClanahan et al. 2007).

In this study, we enriched the water around three common GBR macroalgae species with N or P in a flow through seawater system. We assessed whether short-term nutrient enrichment with N or P increased growth and photosynthetic response of the algae relative to background levels. We made simultaneous measurements of the nucleic acid content as well as the C, N, and P elemental composition in the tissue allowing us to test the GRH in coral reef macroalgae.

## Materials and Methods

### Experimental algae and setup

**Figure 1:**
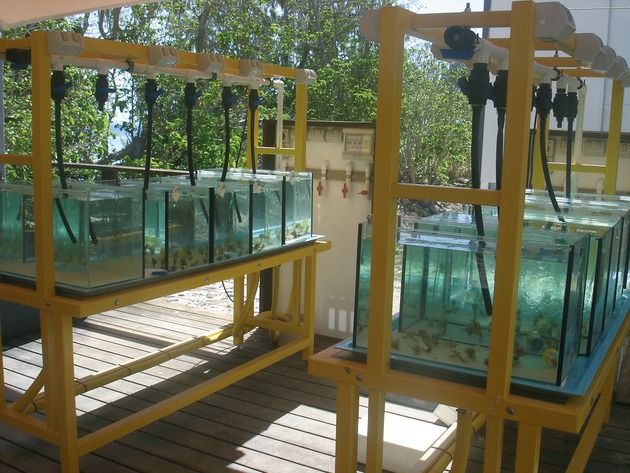
The experimental setup consisting of nine 40-L glass aquaria connected to the flow through seawater system at the Heron Island Research Station. Five preweighted individual thalli from each species (*Caulerpa serrulata*, *Laurencia intricata,* and *Sargassum polyphyllum*) were placed in each of the aquariums. After 24 h, N (as urea) was added in daily pulses to three tanks as fertilizer for a 1-week period, P (as triple superphosphate) was added to three other tanks, while the three remaining tanks were designated as controls.

Individual thalli of *Caulerpa serrulata* (Forsskål) J. Agardh (Chlorophyta), *Laurencia intricata* (Lamouroux) (Rhodophyta), and *Sargassum polyphyllum* (J. Agardh) (Phaeophyceae) were collected from approximately 1 m depth at the Heron Island reef flat (23°26′36″S 151°54′37″E) in December 2009 (austral summer). Following the collection, the algae were spun dry in a salad spinner and weighted. Five individual thalli from each species were placed in each of the nine 40-L glass aquaria (15 thalli per tank) in the outdoor flow through seawater system at the Heron Island Research Station. Reef water flushed through the tanks at a flow rate of approximately 1.5 L/min. Water temperature ranged between 24 and 28°C (diurnal fluctuation). After an acclimation period of 24 h, N (as urea) was added in daily pulses to three tanks as fertilizer for a 1-week period, P (as triple superphosphate) was added to three other tanks, while the three remaining tanks were designated as controls (C, Fig. 1). The pulse of nutrients (fertilizer) was added each morning of the experiment at 06:30 as 40 g of N or 60 g of P in the form of slow release granules in a sealed plastic vial with holes punched in the lid to slow nutrient release into the water. As an N source, Urea (Impact Fertilisers, Australia) was chosen due to its high abundance in floodwaters reaching the GBR in which it makes up more than 50% of the total nitrogen (Wallace et al. 2009). As a P source, we used a triple superphosphate fertilizer (Impact Fertilisers). To the control treatments, an identical plastic container was added but without fertilizer. The algae were placed under a white shade cloth with midday photosynthetic active radiation of 500 μmol/m^2^/s.

In addition to the experimental samples, seven *Laurencia intricata* plants were collected from the reefs around Heron Island (Wistari Lagoon, depth range 1–5 m) during the time of the experiment and analyzed as described for the experimental samples.

### Water sampling procedure

Water samples were taken from each tank daily 1 h after the nutrient addition using 15-mL tubes for the determination of urea, ammonium, and total phosphate concentrations in the water. On the second day of the experiment, additional water samples were taken at 3, 6, and 12 h following the treatment in order to assess the length of the pulse of elevated nutrient concentrations in the aquaria of the flow through seawater system. Water samples were not filtered and were frozen immediately following collection. Urea and ammonia in the thawed water samples were measured using a colorimetric assay (Megazyme International Ireland Ltd., Wicklow, Ireland). Total phosphate in 100 μL aliquots of the water samples was measured in an acidified persulfate autoclave digestion of the organic compounds (Menzel and Corwin 1965) followed by quantification of the released orthophosphate in a colorimetric assay with ammonium molybdate and malachite green (van Veldhoven and Mannaerts 1987).

### Algae sampling procedure

Algae were collected from the tanks after 1 week, a time scale shown to be optimal for algae enrichment experiments (Downing et al. 1999) as after 7 days, depletion of other nutrients and micrograzing responses can begin to confound experimental results. Photosynthetic electron transport rates were measured for each thallus as the maximum relative rate of photosynthetic electron transport (ETR_max,_ Beer et al. 1998) at 1000 μmol/m^2^/s of photosynthetically active radiation, using a portable chlorophyll fluorometer (PAM2100; Heinz Walz, Germany). Photosynthetically active radiation was provided by the halogen light source within the PAM2100. Following photosynthetic measurements, thalli were spun dry in a salad spinner and weighted. Relative growth rates (RGR) were defined as the% increase in mass of each individual thallus relative to the initial measurement. Two thalli of each species from each tank were then snap frozen in liquid nitrogen for RNA:DNA measurements, and the remaining three thalli were frozen at −20°C for analysis of elemental composition. Once at the lab, the frozen tissue at −20°C was lyophilized and ground to a fine powder. Snap frozen tissue remained at −80°C until analysis of RNA and DNA.

### Elemental composition of algal tissue

Dried algal samples were pulverized using a bead mill. Carbon and N concentrations (presented as% mass) of dried algae were determined using mass spectrometry (UC Davis Stable Isotope Facility). The total P concentration (% mass) in the ground material of each thallus was determined using the methods described in Reef et al. (2010). Briefly, an acidified persulfate autoclave digestion of the organic compounds (Menzel and Corwin 1965) was followed by quantification of the released orthophosphate in a colorimetric assay with ammonium molybdate and malachite green (van Veldhoven and Mannaerts 1987).

We measured relative abundance of the stable isotopes of ^13^C and ^15^N in thalli in order to assess inorganic carbon availability and nitrogen sources. Unfortunately, some dried samples were not large enough (<2 mg) to obtain reliable C and N data. The isotopic relative abundances were measured using a PDZ Europa ANCA-GSL elemental analyzer interfaced to a PDZ Europa 20-20 isotope ratio mass spectrometer (Sercon Ltd., Cheshire, UK) at the UC Davis Stable Isotope Facility. The ^13^C delta values are presented relative to the international standard V-PDB. ^15^N delta values are presented relative to air.

### RNA and DNA determination

Frozen samples (1–2 g FW) were ground in liquid N_2_ using a mortar and pestle and homogenized (Tissue-Tearor; BioSpec Products Inc., Bartlesville, OK, USA) in 1 mL of homogenizing buffer as described in Reef et al. (2010). For nucleic acid measurements, we used a fluorescence assay using 2,7-diamino-10-ethyl-9-phenylphenanthridinium bromide (ethidium bromide) and a sequential enzymatic reaction to quantify the contribution of RNA, DNA, and background fluorescence to the total fluorescence of each sample (Reef et al. 2010). The quantities of RNA and DNA were determined for preweighed samples in triplicates in a 96-well plate format. Alongside the samples each plate included RNA and DNA standards at different concentrations to control for interplate variability, to ensure complete hydrolysis of the RNA and DNA in the samples and to determine the fluorescence ratio between RNA and DNA in our experimental system. In this experimental system, the fluorescence yield of RNA was 0.4 that of DNA.

### Statistical analysis

Differences in elemental composition, photosynthetic rates, and isotopic abundance between the treatments and between the algae species were analyzed as a blocked design with nutrient treatment and species as factors, using linear models and analysis of variance (ANOVA). Data were checked for normality by inspecting the linearity of quantile-normal plots, and one data set (RNA per fresh weight) was square root transformed to conform to normality. The relationship between tissue %P and relative growth rate in *Laurencia intricata* was presented as the unimodal Lorentzian bell function where *a* is the height of the peak, *b* is the position of the center of the peak, and *c* is the width of the peak:


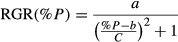


In order to decrease noise, we treated %P in the thallus as an interval variable, with thalli pooled in equally spaced 0.05% ranges from concentrations of 0.10–0.60%. A mean RGR was calculated for each %P interval.

Data analysis was performed using R version 2.13.2 (Team 2011).

## Results

### Water analysis

The fertilizer additions significantly elevated the nutrient concentration in the tanks for a period of >6 h (Table 1). By 12 h following the treatment, nutrient levels had returned to pretreatment levels. The algae in the treatment tanks experienced elevated nutrient treatments during the natural light period.

**Table 1:** Mean concentration (±SD) of N-urea, N-ammonia, and total phosphorus (TP) of water samples taken daily, 1 h following nutrient addition. N and P addition significantly increased N and P concentrations in the N and P treatment tanks, respectively (ANOVA, *F*_(2,15)_ = 6.9, *P* = 0.009 for N and *F*_(2,15)_ = 21.7, *P* < 0.0001 for P)

Treatment	N-Urea (mg/L)	N-Ammonia (mg/L)	TP (μg/L)
C	0.09 (0.07)	<0.01 (0.02)*	21.50 (4.55)
N	3.62 (2.25)	0.08 (0.07)	21.15 (6.96)
P	0.06 (0.07)	<0.01 (0.02)*	281.1 (131.5)

* These values are below the detection limit of the colorimetric test so the ammonia concentration in these treatments could be overestimated.

### Elemental analysis

**Figure 2:**
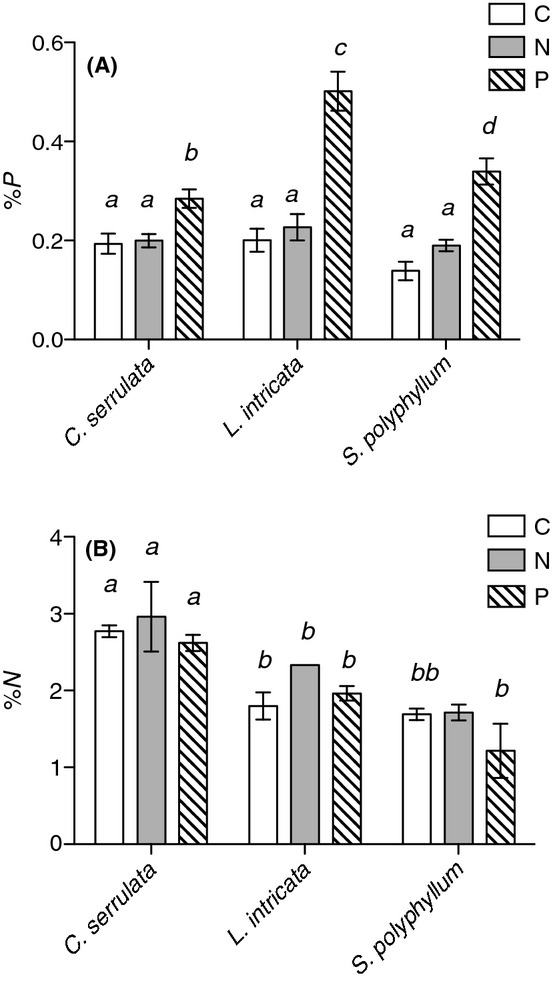
(A) Mean (±SEM) phosphate concentration in algal tissues (mg/g DW) and (B) Mean (±SEM) nitrogen concentration in algal tissues (mg/g DW) of *Caulerpa serrulata*, *Laurencia intricata,* and *Sargassum polyphyllum* after a week in control (open bars), nitrogen-enriched (full bars), and phosphate-enriched (thatched bars) conditions. Different letters indicate significant differences at *P* < 0.05 (Tukey HSD test).

Tissue P concentrations were significantly higher in algae from the P enrichment tanks (ANOVA *F*_(2,78)_ = 56.75, *P* < 0.0001, Fig. 2A). There were also significant differences among species in the control tanks, with *Sargassum polyphyllum* having a significantly lower concentration of P in its tissues than *Caulerpa serrulata* and *Laurencia intricata* (ANOVA *F*_(2,78)_ = 10.4, *P* = 0.0001; LSD post hoc test, *P* = 0.04). Tissue P concentrations of *S. polyphyllum* tissue remained significantly lower than those of *L. intricata* following the P treatment (LSD post hoc, *P* = 0.0002). Nitrogen enrichment had no effect on tissue P concentrations in any of the species.

Tissue N concentrations were significantly different among the three species (*F*_(2,28)_ = 45.4, *P* < 0.0001, Fig. 2B), but nutrient enrichment had no significant effect on N concentrations in the tissues in any of the three species (*F*_(2,28)_ = 3.1946, *P* = 0.063).

N:P ratios were significantly higher for *C. serrulata* compared with the two other species (ANOVA, *F*_(2,28)_ = 11.7, *P* = 0.0003, Table 2). N:P ratios were significantly lower for algae in P-fertilized tanks (*F*_(2,28)_ = 18.7, *P* < 0.0001, Table 2). C:N ratios were significantly higher in *S. polyphyllum* compared with the other species (ANOVA, and *F*_(2,28)_ = 14.64, *P* < 0.0001, Table 2). Nutrient enrichment did not have a significant effect on algal C:N ratios (*F*_(2,28)_ = 2.9, *P* = 0.07). C:P ratios were significantly reduced by the P treatment (ANOVA, *F*_(2,28)_ = 13.2, *P* = 0.0001) and were significantly lower for *L. intricata* compared with the other species (ANOVA, *F*_(2,27)_ = 6.2, *P* = 0.007).

**Table 2:** Mean C:N and C:P and N:P mass ratios (±SD) in tissues of *Caulerpa serrulata*, *Laurencia intricata,* and *Sargassum polyphyllum* under control, nitrogen-enriched, and phosphorous-enriched conditions

	Control	+N	+P
C:N ratio			
*C. serrulata*	12.4 (1.2)	9.9 (0.5)	12.4 (1.1)
*L. intricata*	12.9 (0.7)	10.8 (0.6)	12.8 (3.1)
*S. polyphyllum*	17.6 (1.1)	16.5 (3.0)	27.6 (11.4)
C:P ratio			
*C. serrulata*	209.5 (57.9)	162.5	129.4 (23.6)
*L. intricata*	151.4 (74.7)	156.5	56.1 (9.2)
*S. polyphyllum*	207.9 (56.0)	164.5 (44.3)	110.0 (53.6)
N:P ratio			
*C. serrulata*	17.2 (6.3)	14.8 (1.5)	10.5 (2.2)
*L. intricata*	11.8 (6.1)	14.0	4.8 (0.7)
*S. polyphyllum*	11.8 (3.3)	9.9 (1.8)	3.9 (0.3)

### Isotopic composition of N and C

**Figure 3:**
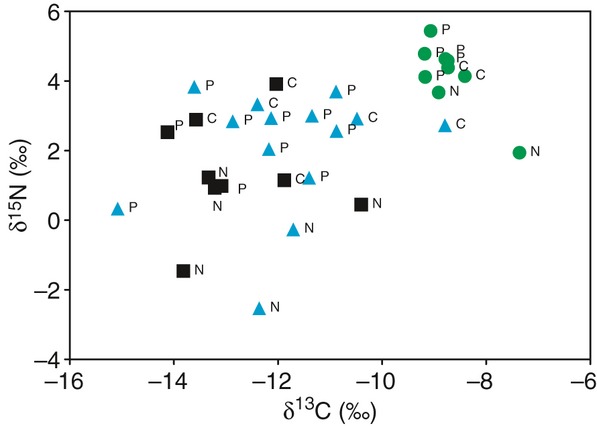
Relationship between the stable isotope composition of N and C in algal thalli that were grown for a week in control (C), nitrogen-enriched (N), or phosphate-enriched (P) conditions. Green circles: *Caulerpa serrulata*, black squares: *Sargassum polyphyllum,* and blue triangles, *Laurencia intricata*.

There were significant differences between the species in δ^15^N (Fig. 3), which showed an interaction with the nutrient enrichment treatment (ANOVA, *F*_(4,28)_ = 4.51, *P* = 0.0092 and subsequent Tukey HSD post hoc tests). Under control conditions, the *C. serrulata* δ^15^N signature was significantly heavier than the other algae (*P* = 0.04). Enrichment with N significantly lowered the δ^15^N values relative to the C and P treatments for all three species (*P* < 0.001); however, due to the small sample size, within-species differences could not be assessed. Enrichment with P did not affect δ^15^N values in any of the species (*P* = 0.96).

The nutrient treatments did not have a significant effect on the abundance of ^13^C isotopes (ANOVA, *F*_(2,30)_ = 1.87, *P* = 0.18, Fig. 3). *C. serrulata* had significantly less negative δ^13^C than the other two species (ANOVA, *F*_(2,30)_ = 26.48, *P* < 0.0001, Fig. 3).

### Nucleic acids

**Figure 4:**
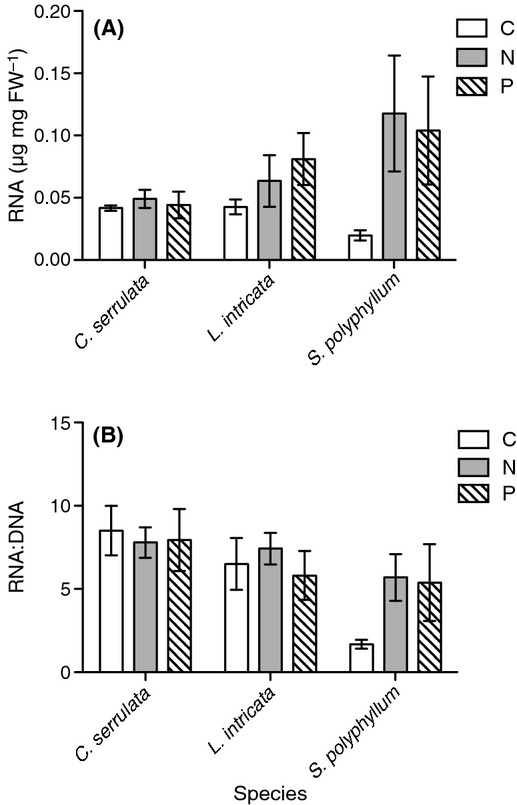
(A) Mean (±SEM) RNA concentration (μg/mg FW) and (B) Mean (±SEM) RNA:DNA ratios in algal tissues of *Caulerpa serrulata*, *Laurencia intricata,* and *Sargassum polyphyllum* after a week in control (open bars), nitrogen-enriched (full bars), and phosphate-enriched (thatched bars) conditions. N and P enrichment had a positive effect on the amount of RNA in algal tissues (ANOVA, *F*_(2,32)_ = 3.63, *P* = 0.04). RNA:DNA ratios varied significantly among species (ANOVA, *F*_(2,34)_ = 4.7, *P* = 0.02).

Both N and P enrichment had a positive effect on the amount of RNA in algal tissues (ANOVA, *F*_(2,32)_ = 3.63, *P* = 0.04, Fig. 4A). RNA concentration on a fresh weight basis was similar among the three different species (ANOVA, *F*_(2,32)_ = 1.06, *P* = 0.36). RNA:DNA ratios did not differ significantly following nutrient addition (ANOVA, *F*_(2,34)_ = 1.16, *P* = 0.32, Fig. 4B) but did vary significantly among species (ANOVA, *F*_(2,34)_ = 4.7, *P* = 0.02).

### Growth

**Figure 5:**
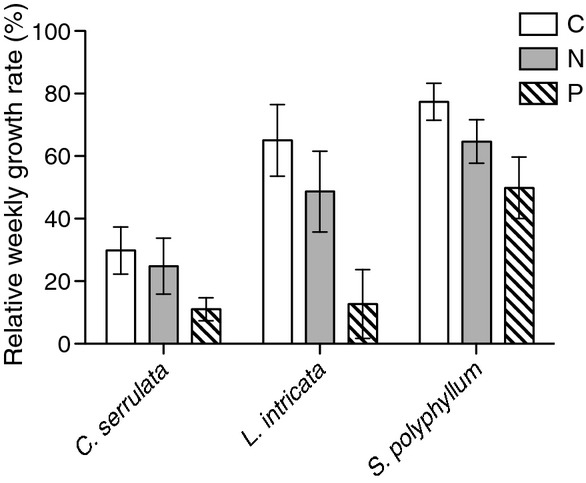
Mean (±SEM) weekly growth relative to initial mass of *Caulerpa serrulata*, *Laurencia intricata,* and *Sargassum polyphyllum* after a week in control (open bars), nitrogen-enriched (full bars), and phosphate-enriched (thatched bars) conditions. All three species had significantly lower growth rates following the addition of P fertilizer relative to both control and N enriched tanks (Tukey HSD, P-C *P* < 0.0003, P-N *P* = 0.03).

Growth rate varied significantly among the species (ANOVA, *F*_(2,118)_ = 12.72, *P* < 0.0001). Growth of *S. polyphyllum* was significantly higher than *C. serrulata*, which had the lowest growth rate of the three species. Growth rates responded significantly to fertilization (*F*_(2,118)_ = 8.79, *P* = 0.0003, Fig. 5). All three species had significantly lower growth rates following the addition of P fertilizer relative to both control and N-enriched tanks (LSD post hoc, P-C *P* < 0.0003, P-N *P* = 0.03). With N fertilizer, algae grew at a similar rate to controls.

**Figure 6:**
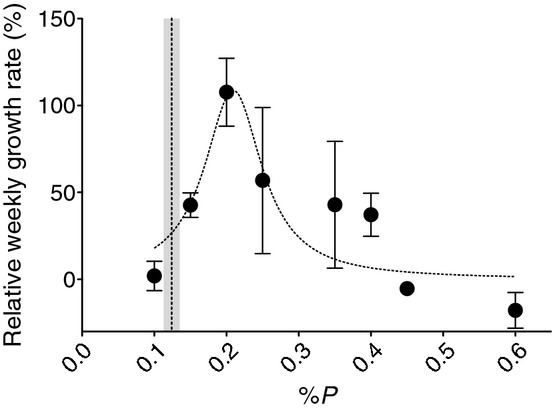
Mean (±SEM) weekly growth rates of *Laurencia intricata* as a function of tissue P concentrations. Means were calculated for thalli grouped based on their P concentrations from 0.10% in 0.05% increments. The relationship between %P and RGR is presented as the unimodal Lorentzian bell function with parameters *a* = 108.7, *b* = 0.21, and *c* = 0.048; *R*^2^ = 0.76. Shaded area corresponds to the mean (±95% CI) tissue P concentrations of seven *L. intricata* plants collected around Heron Island during the time of the experiment.

For *L. intricata*, growth rates varied significantly with tissue %P (ANOVA, *F*_(7,25)_ = 2.975, *P* = 0.029) in a unimodal relationship with peak growth attained in algae with tissue P concentrations of 0.21% (Fig. 6).

### Photosynthetic performance

**Figure 7:**
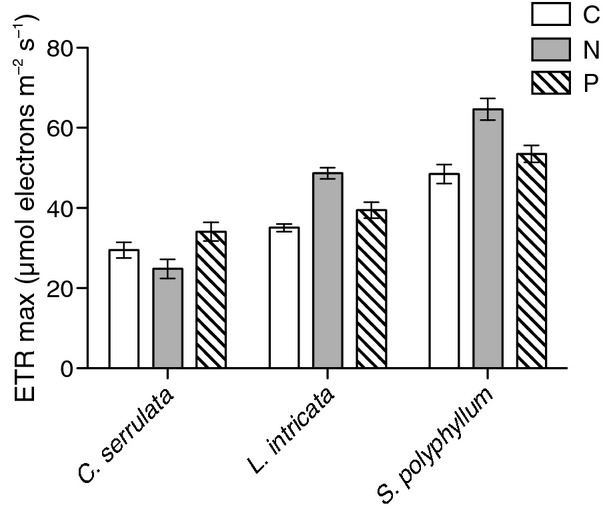
Mean (±SEM) maximum electron transport rates (ETRmax) calculated at 1000 μmol/m^2^/s of photosynthetically active radiation for *Caulerpa serrulata*, *Laurencia intricata,* and *Sargassum polyphyllum* after a week in control (open bars), nitrogen-enriched (full bars) and phosphate-enriched (thatched bars) conditions. Enrichment with N or P resulted in a significant increase in ETR (*F*_(2,114)_ = 5.14, *P* = 0.0074) relative to controls.

Photosynthetic electron transport rates (rETR max) varied significantly among species (ANOVA, *F*_(2,114)_ = 70.23, *P* < 0.0001, Fig. 7) and nutrient treatments (ANOVA, *F*_(2,114)_ = 5.14, *P* < 0.0074, Fig. 7). Enrichment with both N and P resulted in a significant increase in ETR (*F*_(2,114)_ = 5.14, *P* = 0.0074) relative to controls.

## Discussion

Elevating nutrient levels did not result in increased growth rates in any of the macroalgal species in our experiments. However, the algae did respond positively in other ways to nutrient additions, which contradicts the possibility of nutrient toxicity. For example, ETR responded positively to both N and P additions (Fig. 7), RNA concentrations were elevated compared with control in both N and P fertilization for *L. intricata* and *S. polyphyllum* (Fig. 4A) and RNA:DNA ratios increased significantly for *S. polyphyllum* following N (but not P) addition (Fig. 4B). Despite the increases in RNA and P tissue concentrations, growth rates were reduced in the P-fertilized treatment in all three species relative to control (Fig. 5). Therefore, our results do not directly support the growth rate hypothesis, for which correlations among P, RNA, and growth are expected. Reductions in algal growth rate at high nutrient concentrations are well known (Grover 1989; Schaffelke and Klumpp 1998), but the reason for the reduced growth is unclear, as the effect on photosynthesis was positive. Our detailed analysis of growth responses over gradients in P in tissues of *L. intricata* indicates a bell-shaped curve, with growth increasing at intermediate levels of P before declining at high P tissue concentrations. Maximum growth rate was achieved when tissue P concentrations were 0.21% (Fig. 6). The average P concentration in the tissues of seven *L. intricata* plants naturally occurring at Heron Island was significantly lower (0.12%) than that optimal concentration, suggesting that moderate increases in P might enhance algal growth rates.

RNA is widely believed to be the major P constituent in the cell. RNA content was significantly higher in both the N and the P fertilization treatments relative to controls. We could not test for the correlation between RNA content and N or P concentrations in individual thalli, as individuals were frozen for one of the two measurements. However, there were no significant correlations among mean RNA content of a species calculated for each tank and the %P or %N for that tank for any of the three species. Increases in RNA in fertilized treatments compared with controls may be supporting up-regulation of futile cycles, which would require additional metabolic components and other processes requiring protein synthesis that is not coupled to growth (e.g., Britto et al. 2001). Enhanced RNA could therefore be associated with up-regulation of “protective” metabolic processes rather than growth. Nutrient enrichment resulted in higher photosynthetic electron transport rates relative to control in all three species. However, photosynthetic electron transport rates were not correlated with growth over the time frame of this experiment, which indicates higher respiration in the enrichment treatments. The lack of growth response, despite higher rates of photosynthetic electron transport, could be due to diversion of energy to support additional metabolic processes, including futile cycles (photorespiration and ammonium cycling), which could be stimulated by low concentrations of CO_2_ and high concentrations of ammonium (Britto et al. 2001) in the N treatment.

A significant portion of the nutrients, especially P, taken up by the algae can be incorporated into molecules other than RNA, such as phospholipids and polyphosphate granules (Chopin et al. 1997), or stored in intracellular vacuoles (Lundberg et al. 1989). The large increases in P concentrations of tissues with P fertilization without concomitant increases in yield indicate “luxury uptake” of P (an uptake of P that is not associated with biomass production (Gerloff and Krombholz 1966) occurred in all three algal lineages. Luxury uptake was most apparent for *L. intricata*. Unlike most nutrient uptake systems, which follow Michaelis–Menten saturation-type kinetics, many studies suggest that for some algae the phosphate transport system has biphasic kinetics with saturation-type kinetics at low concentrations and a linear uptake response at high external phosphate concentrations. This has been shown for green (Portielje and Lijklema 1994), red (Friedlander and Dawes 1985; Littler et al. 2010) and brown algae (Silkin and Chubchikova 2007). The linear phase most likely represents simple diffusion across the membrane (Lobban and Harrison 1994), although a novel enzymatic system has also been suggested (Silkin and Chubchikova 2007). The nonsaturable P kinetics leading to luxury uptake would be favorable in low nutrient marine environments where high variability in nutrient concentrations and ephemeral micropatches of nutrients are typical (Lehman and Scavia 1982). Macroalgae have most likely evolved to take advantage of transient nutrient pulses (Chapman and Craigie 1977; Raven and Taylor 2002). Our observation that luxury uptake occurred in species from three different algal lineages suggests that biphasic P uptake could be widespread among tropical macroalgae.

The ability to rapidly uptake and store P during a high nutrient pulse for later use during periods of limitation can give a significant advantage in an environment where nutrient availability is patchy, especially in the oligotrophic waters like those surrounding coral reefs. However, it appears that P storage comes at a cost, and above a threshold (0.21%), we observed reduced growth rates (Fig. 6) despite increases in ETR. Our finding of costs of high P concentrations in tissues is similar to a previous study, which showed a similar unimodal response in both growth and survival of animals to tissue P concentrations (Boersma and Elser 2006), expressed as a “stoichiometric knife edge” (Elser et al. 2005). Thus, pollution with high levels of P may be particularly damaging for algae and other organisms evolved in low P environments (e.g., tropical populations; Lovelock et al. 2007).

The small and statistically nonsignificant increase in tissue N concentrations in response to elevated urea/ammonium in the experimental tanks suggest that luxury uptake for storage of N by these three species in this environment does not occur or that there is strong homeostasis of tissue N. The δ^15^N signature of the urea fertilizer is −0.34 (McKee et al. 2002), which is lower than the signature of DIN in coral reef waters (Margalef 1971; Smith et al. 1981; Genin et al. 1995; Heikoop et al. 2000; Szmant 2002). The significant reduction in δ^15^N signatures following the enrichment suggests that uptake of fertilizer N did occur but was not stored (there was a concurrent balanced loss of N), this suggests futile cycling of N. Nitrogen uptake (especially of reduced forms like urea and ammonium) is sensitive to past environmental conditions experienced by organisms (Fujita 1985), higher uptake rates occur when N is limiting growth, and its availability is transient. Thus, our findings that N was not stored by the algae in the high N treatment, despite the likelihood it was taken up, suggest that N is not limiting growth at this location, making luxury uptake of N an unnecessary energetic expenditure. For many algae species, the capacity for luxury uptake of N appears to be much lower than for P (Elrifi and Turpin 1985).

δ^13^C values were similar to those measured previously on the GBR for these genera (Black and Bender 1976) but significantly higher than values previously published for these genera on other coral reefs, and other tropical coral reef macroalgae (Fry et al. 1982) indicating possible inorganic carbon limitation to growth in our experiment (France 1995), which could confound our testing of the growth rate hypothesis (which assumes N or P limitation to growth). Inorganic carbon limitation to growth has been suggested for epilithic algae at both the Gulf of Aqaba and at One Tree Island (Larkum et al. 2003) due to the restriction of mass transfer by the boundary layer around the algae. Wheeler (1980) showed severe carbon limitation to photosynthesis in giant kelp when slow and laminar flow caused slow CO_2_ diffusion rates. The low flow regime at the Heron Island reef flat could therefore result in dissolved inorganic carbon limitation to growth. There were significant differences in δ^13^C between the species; the green algae *C. serrulata* had significantly higher δ^13^C values compared with the red *L. intricata* and brown *S. polyphyllum*.

We found significant interspecific differences in RNA:DNA ratios. RNA:DNA ratios of *S. polyphyllum* were lower than those of the two other species. The RNA:DNA ratios for each species did not correlate with species growth rates. While the RNA:DNA ratio is appropriate for comparing treatments within a species, it might be restricted in interspecific comparisons. The RNA:DNA ratio is a measure for RNA content normalized against the amount of live cells in a sample, presented as DNA content. However, the large differences in DNA content between cells of different taxa (C-value) and the polynucleic nature of the siphonous green *C. serrulata* make interspecific comparisons in RNA:DNA ratios difficult. However, even when normalized against their C-values: 0.11, 0.24, and 0.78 pg DNA per unreplicated gamete cell for *C. serrulata*, *S. polyphyllum,* and *L. intricata*, respectively (Kapraun 2005), *C. serrulata* and *S. polyphyllum* RNA:DNA values are significantly lower than those of *L. intricata,* which does not correspond to the ranking of growth rates for those species.

The growth rate hypothesis (GRH) proposes that faster growing organisms require an overall greater P content, which is associated with more rRNA and increased capacity for protein synthesis. The astounding ability of algae to uptake and store nutrients in a manner uncoupled from growth, and even at the expense of current growth, is probably the main reason we found very limited support for the GRH in this study. We did find support for the “stoichiometric knife edge” hypothesis, where high levels of P result in negative growth effects in algae. The loose regulation of tissue stoichiometry has a clear adaptive advantage in the nutrient poor environment of coral reefs as luxury uptake enables algae to rapidly exploit nutrient rich patches and store them for future growth, which could increase competitive ability. In these coral reef algae, the benefits of investing in P uptake and storage for future use outweigh the advantages of maintaining the stoichiometry that would facilitate maximum growth rates (Fig. 6). However, several studies have shown repressed growth rates, survivorship, and fitness in animals feeding on P rich food (reviewed in Boersma and Elser 2006). Thus, maintenance of high P concentrations in the algal tissues can have negative flow-on effects on herbivores, which could lead to losses in ecosystem resilience (Burkepile and Hay 2006).
